# Etymologia: Mimivirus

**DOI:** 10.3201/eid2610.ET2610

**Published:** 2020-10

**Authors:** Clyde Partin

**Affiliations:** Emory University School of Medicine, Atlanta, Georgia, USA

**Keywords:** etymologia, mimivirus, viruses, bacteria, Acanthamoeba polyphaga, amoeba

## Mimivirus [mĭm¢ĭ-vī¢rǝs]

If virus (Latin: slimy) challenges the definition of what constitutes life, the DNA mimivirus tests how we define virus. This unidentifiable “bacterium” infecting *Acanthamoeba polyphaga *(Figure), was isolated in 1992 from a hospital cooling tower in Bradford, England. Thus, the original name was *Bradfordcoccus*, and it was considered a culprit for a pneumonia outbreak at this hospital.

**Figure:**
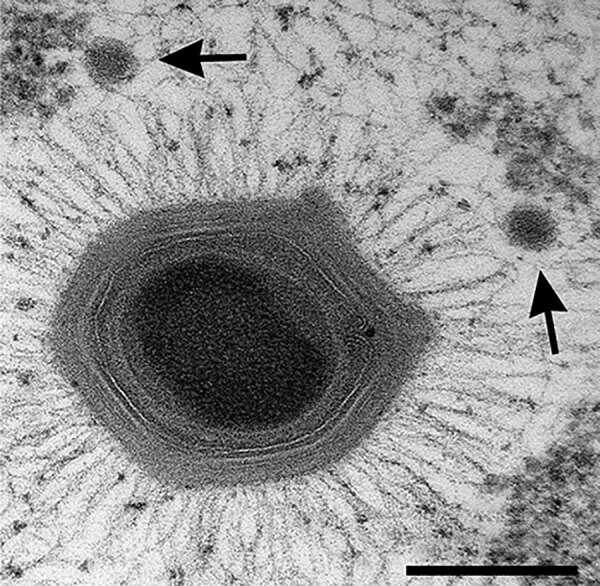
*Acanthamoeba polyphaga* mimivirus, with two satellite Sputnik virophages (arrows). Thin-section electron microscopy courtesy of J.Y. Bou Khalil and B. La Scola, IHU Mediterranée Infection, France.

Researchers brought samples to Didier Raoult and colleagues at Aix-Marseille University, who eventually identified this “bacterium” as a novel virus in 2003. The physical size, genomic content, and ability of the outer protein coat to stain gram positive, thus mimicking (Latin: imitate) prokaryotic bacteria, indicated that this pathogen might be a bacterium.

Raoult initially claimed that the moniker meant “mimicking microbe” but later sheepishly recounted a childhood memory about his father, a physician–scientist, who created stories to explain evolution. Featured prominently in these whimsical narratives was an anthropomorphic character named “Mimi the amoeba.”
